# Association between cholinesterase activity and critical illness brain dysfunction

**DOI:** 10.1186/s13054-022-04260-1

**Published:** 2022-12-06

**Authors:** Christopher G. Hughes, Christina S. Boncyk, Benjamin Fedeles, Pratik P. Pandharipande, Wencong Chen, Mayur B. Patel, Nathan E. Brummel, James C. Jackson, Rameela Raman, E. Wesley Ely, Timothy D. Girard

**Affiliations:** 1 Department of Anesthesiology, Division of Anesthesiology Critical Care Medicine and Critical Illness, Brain Dysfunction, and Survivorship Center, Vanderbilt University Medical Center, 1211 21st Ave. South, 422 MAB, Nashville, TN 37212 USA; 2 United States Air Force, Washington, USA; 3 Department of Anesthesiology, University of Maryland School of Medicine, Baltimore, MD USA; 4 Departments of Anesthesiology and Surgery, Division of Anesthesiology Critical Care Medicine and Critical Illness, Brain Dysfunction, and Survivorship Center, Vanderbilt University Medical Center, Nashville, TN USA; 5 Department of Biostatistics, Vanderbilt University School of Medicine, Nashville, TN USA; 6 Critical Illness, Brain Dysfunction, and Survivorship Center, Vanderbilt University Medical Center, Nashville, TN USA; 7 Department of Surgery, Division of Acute Care Surgery and Critical Illness, Brain Dysfunction, and Survivorship Center, Vanderbilt University Medical Center, Nashville, TN USA; 8 Nashville Veterans Affairs Medical Center, Tennessee Valley Healthcare System, Nashville, TN USA; 9 Division of Allergy, Critical Care and Sleep Medicine, Department of Medicine, The Ohio State University Wexner Medical Center, Columbus, OH USA; 10 Department of Medicine, Division of Pulmonary and Critical Care Medicine and Critical Illness, Brain Dysfunction, and Survivorship Center, Vanderbilt University Medical Center, Nashville, TN USA; 11 Geriatric Research, Education and Clinical Center Service, Nashville Veterans Affairs Medical Center, Tennessee Valley Healthcare System, Nashville, TN USA; 12 Department of Critical Care Medicine and Clinical Research, Investigation, and Systems Modeling of Acute Illnesses Center, University of Pittsburgh, Pittsburgh, PA USA

**Keywords:** Acetylcholinesterase, Butyrylcholinesterase, Delirium, Cognitive impairment, Critical illness

## Abstract

**Background:**

Delirium is a frequent manifestation of acute brain dysfunction and is associated with cognitive impairment. The hypothesized mechanism of brain dysfunction during critical illness is centered on neuroinflammation, regulated in part by the cholinergic system. Point-of-care serum cholinesterase enzyme activity measurements serve as a real-time index of cholinergic activity. We hypothesized that cholinesterase activity during critical illness would be associated with delirium in the intensive care unit (ICU) and cognitive impairment after discharge.

**Methods:**

We enrolled adults with respiratory failure and/or shock and measured plasma acetylcholinesterase (AChE) and butyrylcholinesterase (BChE) activity on days 1, 3, 5, and 7 after enrollment. AChE values were also normalized per gram of hemoglobin (AChE/Hgb). We assessed for coma and delirium twice daily using the Richmond Agitation Sedation Scale and the Confusion Assessment Method for the ICU to evaluate daily mental status (delirium, coma, normal) and days alive without delirium or coma. Cognitive impairment, disability, and health-related quality of life were assessed at up to 6 months post-discharge. We used multivariable regression to determine whether AChE, AChE/Hgb, and BChE activity were associated with outcomes after adjusting for relevant covariates.

**Results:**

We included 272 critically ill patients who were a median (IQR) age 56 (39–67) years and had a median Sequential Organ Failure Assessment score at enrollment of 8 (5–11). Higher daily AChE levels were associated with increased odds of being delirious versus normal mental status on the same day (Odds Ratio [95% Confidence Interval] 1.64 [1.11, 2.43]; *P* = 0.045). AChE/Hgb and BChE activity levels were not associated with delirious mental status. Lower enrollment BChE was associated with fewer days alive without delirium or coma (*P* = 0.048). AChE, AChE/Hgb, and BChE levels were not significantly associated with cognitive impairment, disability, or quality of life after discharge.

**Conclusion:**

Cholinesterase activity during critical illness is associated with delirium but not with outcomes after discharge, findings that may reflect mechanisms of acute brain organ dysfunction.

*Trial Registration: *NCT03098472. Registered 31 March 2017.

**Supplementary Information:**

The online version contains supplementary material available at 10.1186/s13054-022-04260-1.

## Background

Delirium is a manifestation of acute brain dysfunction involving impairments in attention and cognition that affects up to half of older hospitalized patients[1, 2] and 50%-75% of critically ill patients [3], such that millions of patients worldwide experience this acute threat to their health and wellbeing every year. Delirium in ICU patients results in enormous financial and societal burdens due to its relationship with longer ICU and hospital stays [4], increased health care costs [5], higher short- and long-term mortality [6–8], and increased risk of long-term cognitive impairment [9, 10], an acquired disorder that for many patients is akin to dementia.

Proinflammatory markers are elevated during ICU delirium [11, 12], but the nature of the relationship between inflammation—which is present in the majority of critically ill patients—and delirium remains poorly understand. Acute neuroinflammatory changes may lead to delirium in the acute setting and chronic neuronal alterations manifesting as cognitive impairment in the long-term [13–15]. A widely held hypothesis proposes that inflammation is regulated by the cholinergic system, and that this interaction plays a pivotal role in whether delirium develops in the setting of acute illness [14, 16]. Thus, the ability to measure cholinergic system activity may provide insight into critically ill patients at particularly high risk for acute and long-term brain dysfunction.

The primary neurotransmitter of the cholinergic system is acetylcholine, which can’t be measured directly in clinical settings. The activity of acetylcholine is regulated by the enzymes acetylcholinesterase (AChE), primarily found in synapses and red blood cell membranes, and butyrylcholinesterase (BChE), primarily found in plasma. Changes in the activity of these enzymes, which can be measured in whole blood using point-of-care testing, may reflect altered regulation of circulating acetylcholine and inflammation. Increased AChE activity has been associated with increased risk of postoperative delirium [17] and with major neurocognitive disorder [18]. Decreased BChE activity has been associated with increased inflammation [19–21], increased risk of postoperative delirium [17, 22–24], and dementia [25, 26]. Nevertheless, the activity of these enzymes has not been evaluated in larger multidisciplinary critically ill cohorts where delirium and cognitive impairment after discharge are common.

We, therefore, conducted a prospective cohort study to investigate whether AChE, AChE normalized per gram of hemoglobin (AChE/Hgb), and BChE activities measured using point-of-care testing are associated with acute brain dysfunction (i.e., delirium and coma) during critical illness and whether they are predictive of long-term cognitive impairment, disability, and health-related quality of life in survivors of critical illness.

## Methods

### Study design and population

We conducted the Cholinesterase Activity and DeliriUm during Critical illness Study (CADUCeuS) (NCT03098472) at Vanderbilt University Medical Center from May 2017 until January 2020. The study protocol was approved by the Vanderbilt Institutional Review Board. We included adult patients enrolled in prospective studies within the Critical Illness, Brain Dysfunction, and Survivorship Center who were admitted to the medical, surgical, or trauma ICU with respiratory failure (mechanical ventilation or non-invasive positive pressure ventilation) and/or shock requiring vasopressors unless they met the following exclusion criteria: expected death within 24 h or planned transition to comfort measures; active substance abuse, psychotic disorder, or homelessness without a secondary contact which would make long-term follow-up difficult; blindness or deafness which prevents assessments; inability to obtain informed consent from the patient or surrogate.

### Exposures

We collected 10 µL of whole blood in the mornings of days 1 (study enrollment), 3, 5, and 7 while in the hospital to measure AChE and BChE activities using the validated ChE Check device (LISA-CHE, Dr. F. Köhler Chemie [DFKC] Bensheim, Germany), a point-of-care device that reliably measures AChE and BChE activities in whole blood. These collection intervals were chosen to capture trends in enzyme activity from presentation of critical illness through resolution based on historical cohorts within our institution. Enzyme activity was measured in U/L. In addition to measuring and assessing relationships for AChE activity, we also normalized the AChE activity per gram of hemoglobin (AChE/Hgb) as AChE is found in synapses and on red blood cell membranes.

### Outcomes

We assessed patients for delirium and/or coma twice daily until ICU discharge and once daily after ICU discharge. To determine the level of arousal, we used the Richmond Agitation-Sedation Scale (RASS) [27], and to assess for delirium, we used the Confusion Assessment Method for the ICU (CAM-ICU) [28]. We considered coma present if the RASS was − 4 (responsive to physical stimulus only) or − 5 (completely unresponsive). We considered delirium present if the patient was not comatose (i.e., had a RASS of − 3 or more) and was CAM-ICU positive. We considered a patient to have a ‘normal’ mental assessment if neither coma nor delirium were present.

Neuropsychology professionals blinded to each participant’s hospital course and biomarker data assessed participants for cognitive impairment, disability, and quality of life 3–6 months after hospital discharge. We assessed cognitive function with the Repeatable Battery for the Assessment of Neuropsychological Status (RBANS) [29] and Trail Making Test Part B (Trails B) [30] or with the Telephone Interview for Cognitive Status (TICS) [31] and a validated telephone cognitive battery [32] depending on the co-enrolled study. We defined cognitive impairment present if scores were ≥ 2 standard deviations below normal in one test or ≥ 1.5 standard deviations below normal in any two tests, similar to prior publications [9, 33, 34]. We assessed disability status with the Katz Activities of Daily Living (ADLs) [35] and the Functional Activities Questionnaire (FAQ) [36] and defined presence of disability as ADL score ≥ 1 or FAQ ≥ 2. Finally, we assessed health-related quality of life with the EQ-5D questionnaire [37]. For additional information on these assessments, please see Additional file 1: Supplemental Appendix.

### Covariates

Through medical record review and patient and surrogate interview, we collected demographic data upon enrollment and hospital course data from admission until discharge, death, or a maximum of 30 days after enrollment. We chose the following covariates a priori based on clinical judgment and prior research: age; disability on enrollment; education level (below bachelor’s degree, bachelor’s degree and above); comorbid disease burden per the Charlson comorbidity index; severity of illness per the modified Sequential Organ Failure Assessment Score (which excluded Glasgow Coma Scale since we accounted for coma and delirium separately); sepsis on enrollment; ICU type (medical, surgical, trauma); and mechanical ventilation.

### Statistical analysis

**Fig. 1:**
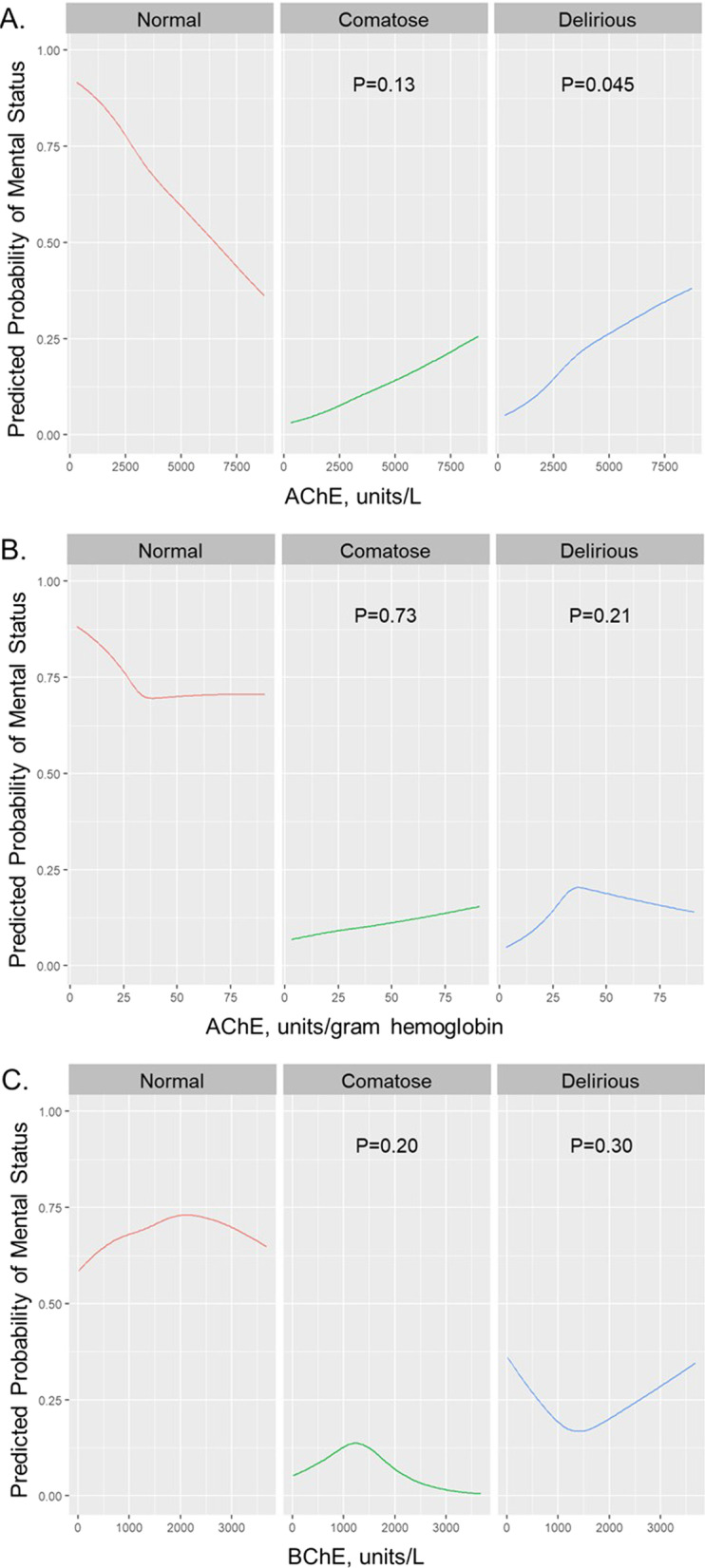
Daily Cholinesterase Enzyme Activity and Daily Mental Status. The predicted probabilities of normal mental status, comatose mental status, and delirious mental status for acetylcholinesterase (AChE, panel** A**), acetylcholinesterase per hemoglobin (AChE/Hgb, panel** B**), and butyrylcholinesterase (BChE, panel** C**) activity levels are displayed. The *P* values represent the independent associations of the enzyme value on the odds of comatose vs. normal mental status or delirious vs. normal mental status. Greater AChE enzyme activity levels had decreased odds of normal mental status and were significantly associated with a higher odds of delirious mental status on the same day (*P* = 0.045) but not comatose mental status (*P* = 0.13). AChE/Hgb and BChE activity levels did not show clear relationships with normal mental status and were not significantly associated with odds of comatose or delirious mental status

We used separate multivariable regression models to determine whether AChE, AChE/Hgb, and BChE activity were independently associated with outcomes. To assess the association of daily enzyme activities with acute brain dysfunction, we performed multinomial logistic regressions to analyze the association between the daily enzyme activity levels (i.e., day 1, 3, 5, 7) and the same day’s mental status categorized as normal, delirium (if one or more assessment was CAM-ICU positive), or coma (if both assessments were RASS −4 of −5), adjusting for the aforementioned covariates (using the same day SOFA score, ICU type, and mechanical ventilation status) and the prior day’s mental status (i.e., normal, delirium, coma). We fit a multinomial logistic regression model given the nominal outcomes (i.e., normal brain function, delirium, coma). Similar to a logistic regression model, the results from this model can be presented both as an odds ratio and the predicted probability of an event happening. We choose to present the results as predicted probabilities to clearly visualize the relationship between enzyme activity levels and mental status in Fig. 1. The odds ratios from these models comparing enzyme activity level and outcomes are also provided in Additional file 1: Figs. S7 (odds of coma vs. normal) and S8 (odds of delirium vs. normal). We also performed proportional odds logistic regressions to analyze the association of the enzyme activity level on day 1 with the number of calendar days alive without delirium or coma over 14 days. This accounts for bias due to death, and higher numbers equate to less acute brain dysfunction (i.e., more days alive and free of delirium or coma). We considered days after the day of hospital discharge as without delirium or coma until the end of 14-day period or death, whichever came first.

To assess the association of enzyme activities with long-term cognitive impairment, we performed separate multivariable logistic regressions analyzing each enzyme’s day 1 value and mean value during the hospital stay and the presence of cognitive impairment (yes/no), adjusting for the aforementioned covariates (using mean SOFA score) except for ICU type and mechanical ventilation to avoid overfitting. We performed similar multivariable logistic regressions analyzing each enzyme’s day 1 value and mean value and the presence of disability (yes/no). Finally, we performed separate multivariable proportional odds logistic regressions analyzing each enzyme’s day 1 value and mean value and the EQ-5D index score, adjusting for the aforementioned covariates (using mean SOFA score and mechanical ventilation duration) with the addition of delirium duration.

We incorporated restricted cubic splines for continuous variables into the models based on the distribution. Prior to modeling, we performed redundancy analyses to detect multicollinearity of covariates. We used R software version 3.6.3 for all the analyses.

### Role of the funding source

For this investigator-initiated study, the sponsor provided a research grant and the ChE Check device. The sponsor had no role in study design, data collection, data analysis, data interpretation, or writing of the report.

## Results

We enrolled 272 patients into the study who were a median (IQR) age 56 (39–67) years and had a median Sequential Organ Failure Assessment score at enrollment of 8 (5–11), 176 of whom survived to discharge and had follow-up assessments. Patient characteristics from the overall cohort are presented in Table 1 and for those who participated in follow-up in Additional file 1: Table S1. Overall, there was a balance between trauma, medical, and surgical ICU patients with a high severity of illness, including 55% with sepsis and 69% on mechanical ventilation. Fifteen percent of the cohort died within the hospital, and 23% died within 90 days of enrollment. The median days alive and free of delirium or coma was 11 (2–13) out of 14 days. We found that 47% of the survivors had scores indicative of cognitive impairment, and 40% had evidence of disability (Table 1). Scores on the cognitive, disability, and health-related quality of life assessments are provided in Additional file 1: Table S2.

**Table 1:** Characteristics and outcomes of study population

Characteristic*	*N* = 272
Age at enrollment, years	56 (39–67)
Male sex, *N* (%)	151 (56%)
Body mass index (kg/m^2^)	28.7 (24.4–33.7)
Education, *N* (%)	
• Below bachelor’s degree	223 (83%)
• Bachelor’s degree and above	47 (17%)
Charlson Comorbidity Index at enrollment	1 (0–2)
Disability present at enrollment, *N* (%)	56 (21%)
Sepsis at enrollment, *N* (%)	149 (55%)
Mechanical ventilation at enrollment, *N* (%)	187 (69%)
SOFA score at enrollment	8 (5–11)
ICU type, *N* (%)	
• Medical	93 (34%)
• Surgical	63 (23%)
• Trauma	116 (43%)
ICU length of stay (days)	3.8 (1.4–8.8)
Hospital length of stay (days)	9.1 (5.1–16.0)
Days with delirium	1.0 (0.0–4.0)
Days with coma	0.0 (0.0–1.0)
Days alive and free of delirium or coma†	11.0 (2.0–13.0)
In-hospital mortality, *N* (%)	42 (15%)
90-day mortality, *N* (%)	63 (23%)
Cognitive impairment present at follow-up (*N* = 154), *N* (%)	72 (47%)
Disability present at follow-up (*N* = 176), *N* (%)	71 (40%)
EQ-5D index score at follow-up (*N* = 170)	0.7 (0.4–0.8)

*Median (interquartile range) or* N* (percentage)

^†^Days alive and free of delirium or coma in the 14 days from enrollment

Participant characteristics and outcomes of the cohort, including acute brain dysfunction, cognitive impairment, disability, and mortality, are displayed. Cognitive impairment was assessed with the Repeatable Battery for the Assessment of Neuropsychological Status [29] and Trail Making Test Part B [30] or with the Telephone Interview for Cognitive Status [31] and a validated telephone cognitive battery [32]. Disability was assessed with the Katz ADL [7] and the Functional Activities Questionnaire [8]

*ICU* intensive care unit, *SOFA* Sequential Organ Failure Assessment

### Cholinesterase enzyme activity

Table 2 displays the median (interquartile range) of the enzyme activity values throughout the study period and on days with normal mental status, comatose mental status, and delirium mental status. Enzyme activity levels for the cohort are displayed graphically in Additional file 1: Figs. S1–S6. In general, AChE and BChE activity levels decreased from initial measurement through the hospital stay, with the largest decrease from day 1 to day 3. AChE/Hgb activity levels remained similar from day 1 to 7. Median AChE activity levels appeared slightly lower on comatose and delirious days compared to days with normal mental status, though not when normalized to Hgb. Median BChE activity levels were more noticeably lower on comatose and delirious days than normal mental status.

**Table 2:** Median cholinesterase enzyme activity levels

Period	N	AChE^*^	AChE/Hgb^*^	BChE^*^
Day 1	253	3382 (2823–4108)	32.2 (29.4–35.2)	1382 (1068–1686)
Day 3	215	3128 (2560–3845)	32.0 (29.6–35.6)	1247 (968–1609)
Day 5	157	3123 (2713–3737)	32.0 (29.4–34.4)	1298 (926–1585)
Day 7	43	3109 (2588–3756)	32.9 (29.8–38.6)	1144 (860–1293)
Sum Day 1–7	668	3237 (2683–3933)	32.1 (29.5–35.4)	1271 (989–1630)
Normal mental status	328	3288 (2732–4076)	32.3 (29.7–35.0)	1368 (1101–1695)
Comatose mental status	123	3026 (2467–3689)	31.4 (28.7–35.1)	1214 (903–1417)
Delirious mental status	212	3237 (2713–3889)	32.1 (29.6–35.8)	1212 (880–1674)

*Median (interquartile range) enzyme activity level in Units/L for AChE and BChE and in Units/gram hemoglobin for AChE/Hgb

The median acetylcholinesterase (AChE), acetylcholinesterase per hemoglobin (AChE/Hgb), and butyrylcholinesterase (BChE) activity levels on days 1, 3, 5, and 7 are displayed. The median activity levels overall, along with activity levels on days with normal, comatose, and delirious mental status are also displayed

### Acute brain dysfunction

To assess the independent association of enzyme activities with acute brain dysfunction, we first analyzed the association between the daily enzyme activity value and the same day’s mental status, adjusting for covariates. Figure 1 displays the relationships of enzyme activity levels and the probability of normal, comatose, or delirious mental status. As shown in the figure, higher AChE enzyme activity levels were associated with a decreased odds of normal mental status and a higher odds of delirious status on the same day (*P* = 0.045), but not comatose mental status (*P* = 0.13). The odds of coma and delirium comparing the 75th versus 25th percentile values of the enzyme activity are displayed in Additional file 1: Figs. S7 and S8, respectively. Patients with AChE activity at the 75th percentile, for example, would have on average a 64% increased odds of developing delirium compared to those with values at the 25th percentile. AChE/Hgb, however, and BChE activity levels did not show clear relationships with normal mental status and were not significantly associated with odds of comatose or delirious mental status.

**Fig. 2:**
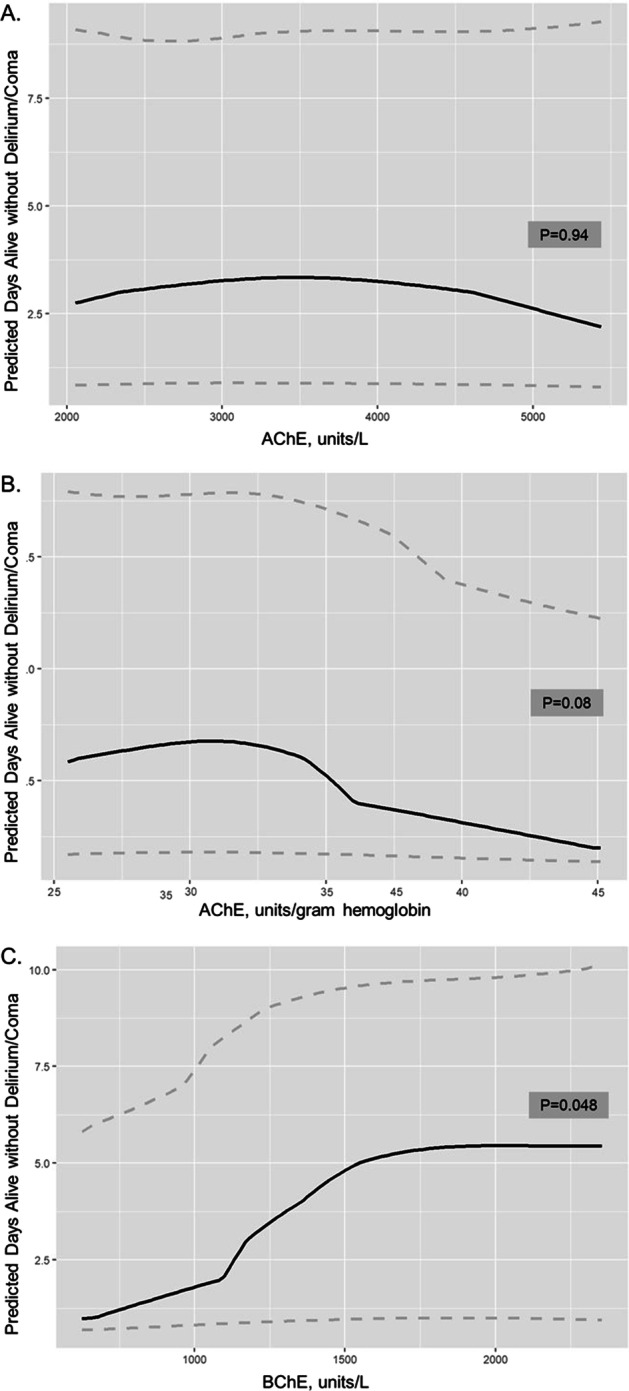
Enrollment Cholinesterase Enzyme Activity and Days Alive Without Delirium or Coma. The predicted days alive without delirium or coma in 14 days from enrollment (day 1) acetylcholinesterase (AChE, panel** A**), acetylcholinesterase per hemoglobin (AChE/Hgb, panel** B**), and butyrylcholinesterase (BChE, panel** C**) activity levels are displayed. The grey dashed lines represent the 95% confidence intervals. Lower enrollment BChE activity levels were associated with fewer days alive without delirium or coma over the following 14 days (*P* = 0.048), indicating worse acute brain dysfunction. Enrollment AChE and AChE/Hgb activity were not significantly associated with days alive without delirium or coma

We next analyzed the association of each enzyme activity value on enrollment (day 1) with the number of calendar days alive without delirium or coma over 14 days, adjusting for relevant covariates. Figure 2 displays these relationships. Enrollment AChE and AChE/Hgb activity were not significantly associated with days alive without delirium or coma. However, lower enrollment BChE activity levels were associated with fewer days alive without delirium or coma over the following 14 days (*P* = 0.048), indicating worse acute brain dysfunction. Additional file 1: Fig. S9 displays the odds of greater days alive without delirium or coma by the 75th versus 25th percentile of enzyme values. Patients with BChE activity at the 75th percentile, for example, would, on average, have a 44% increased odds of having more days alive without delirium or coma (favorable outcome, indicating less brain dysfunction) compared to those with activity at the 25th percentile, supporting that lower BChE values are associated with worse acute brain dysfunction.

### Cognitive and functional impairments

**Fig. 3:**
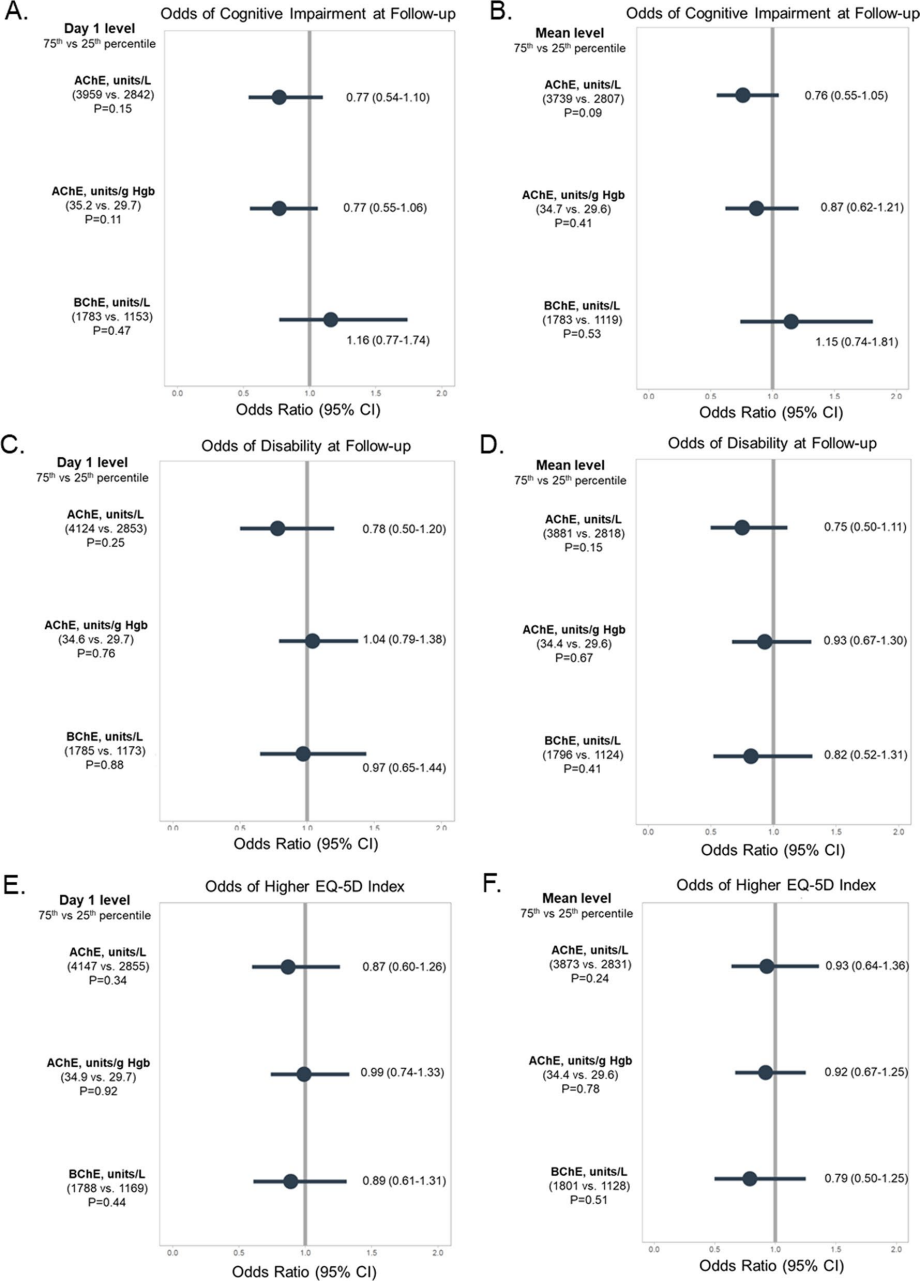
Cholinesterase Enzyme Activity and Neuropsychological Outcomes. The odds ratio (95% confidence interval) of cognitive impairment (panels** A** and** B**), disability in activities of daily living (panels** C** and** D**), and better health-related quality of life on the EQ-5D (panels** E** and** F**) for the 75th percentile acetylcholinesterase (AChE), acetylcholinesterase per hemoglobin (AChE/Hgb), and butyrylcholinesterase (BChE) activity levels compared to the 25th percentile enzyme levels are displayed. Also listed are the *P* values of the overall relationships. We did not find any statistically significant associations between either enrollment (day 1) or median AChE, AChE/Hgb, or BChE activity levels and cognitive or functional impairments up to 6 months after discharge

We assessed the independent associations of enzyme activity values on enrollment (day 1) and mean activity values during the hospital stay with cognitive impairment, disability, and quality of life 3–6 months after hospital discharge, adjusting for relevant covariates. We did not find any statistically significant associations between either enrollment or mean AChE, AChE/Hgb, or BChE activity levels and outcomes up to 6 months after discharge. Figure 3 displays the odds of cognitive impairment (panels A and B), odds of disability in activities of daily living (panels C and D), and the odds of better health-related quality of life on the EQ-5D (panels E and F) for the 75th percentile enzyme activity level compared to the 25th percentile enzyme level, along with listing the P-values of the overall relationships. Additional file 1: Figs. S10–S12 display the enzyme activity levels and probability of cognitive impairment, probability of disability, and EQ-5D index score at follow-up, respectively.

## Discussion

In this prospective cohort study that measured AChE, AChE/Hgb, and BChE enzyme activity levels using point-of-care testing in critically ill patients over several days, we found that higher AChE activity on a given day was associated with an increased odds of being delirious, though not when normalized to Hgb. Additionally, lower BChE activity was associated with fewer days alive without delirium or coma. We did not find associations between AChE, AChE/Hgb, or BChE activity levels with cognitive impairment, disability, or health-related quality of life up to 6 months after hospital discharge.

There remains a lot of interest in elucidating mechanisms for acute brain dysfunction (i.e., delirium and coma) during critical illness so that tailored therapeutics and patient management protocols can be developed. Moreover, the mechanisms linking delirium during critical illness and longer-term cognitive impairment in survivors remain unclear [38]. The majority of recent animal and human research has focused on systemic insults (e.g., sepsis) leading to inflammatory signaling through the blood brain barrier, resulting in microglial activation, neuroinflammation, and neurotransmitter alterations that present as acute brain dysfunction and then result in chronic neuronal alterations and atrophy manifesting as cognitive impairment in the long-term [13–15, 39–41]. Increasing data support the role of the cholinergic system in regulating the inflammatory response [14, 16, 42]. During inflammatory states, cholinergic stimulation blocks endothelial activation and leukocyte recruitment [43, 44], and a decrease of cerebral acetylcholine triggers increased secretion of IL-1β, IL-6, TNFα, and other inflammatory markers [45]. Cholinergic loss can induce microglia priming, leading to central nervous system amplification of peripheral inflammatory signals [13]. Indeed, a number of anticholinergic agents are known to induce delirium [46], and early studies found serum anticholinergic activity was associated with delirium [47, 48]. There is also an overlap of cholinergic pathways and neuroimaging lesions in delirium [49]. Acetylcholine deficits have been associated with executive dysfunction and memory issues, with anticholinergic medications associated with changes in cognition [50]. Pre-clinical data support that increased cholinergic stimulation can reduce brain inflammation and microglial activation to improve cognitive responses [51] and physostigmine (a cholinesterase inhibitor) produces an anti-inflammatory effect in the brain with increasing acetylcholine levels [45, 52]. While cholinesterase inhibitors to increase acetylcholine availability have not yet shown use in preventing or treating delirium [53, 54], they are used for treatment of Alzheimer’s disease and for patients with anticholinergic overdose and delirium [55, 56].

Measuring acetylcholine levels is not clinically feasible, but activity levels of AChE and BChE can be measured, including through the use of portable point-of-care testing. Additionally, AChE activity can be readily normalized to account for AChE found on red blood cells. Measuring activity levels may assist in guiding potential therapies for delirium in appropriate patients. It appears AChE activity, which is primarily found in synapses and red blood cell membranes, may be linked to more chronic cholinergic activity and neurologic deficit or vulnerability. Increased AChE activity, potentially indicating low acetylcholine availability in the synapses, has been associated with increased risk of postoperative delirium [17] and with major neurocognitive disorder [18]. In this cohort study of critically ill patients, we found that increased AChE activity was independently associated with increased odds of delirium (though not when normalized to Hgb levels) but was not significantly associated with cognitive impairment, or other impairments, after discharge. Thus, this potential vulnerability for acute brain dysfunction did not translate to long-term deficits.

BChE activity, which is primarily found in plasma, correlates with increasing inflammation, greater illness severity, and worse outcomes in critically ill patients [19–21]. As such, decreasing levels appear to be a marker of acute stress, inflammation, and potentially neuroinflammation. Decreased BChE activity has been associated with increased risk of postoperative delirium in several studies [17, 22–24]—though this finding is not universal [57]—and has been associated with dementia [25, 26]. We found that decreased BChE activity early on in the critical illness course was independently associated worse acute brain dysfunction as shown by lower days alive and free of delirium or coma. This provides support of the link between decreased BChE activity, increased inflammation, and increased acute brain dysfunction, yet its activity does not subsequently predict longer lasting impairments in survivors.

Our study had several strengths and limitations. The cohort consisted of medical, surgical, and trauma patients, increasing generalizability. Trained research nurses performed RASS and CAM-ICU assessments, and trained neuropsychology professionals performed follow-up assessments of cognition, disability, and health-related quality of life. We used prospectively collected data to evaluate potential relationships between enzyme activities and both acute and long-term brain dysfunction. Our limitations include use of AChE and BChE activities as markers of the cholinergic system as acetylcholine is not directly measurable. Similarly, as direct measurement of central nervous system inflammatory processes is not feasible in many patients, we used more readily available plasma markers as surrogates of central nervous system pathology. Our samples were collected on frequent, designated study days in the mornings; we did not, however, specify exact timing of sample collection, which may potentially exhibit diurnal variances with ACh activity [58, 59]. Additionally, we did not collect daily administration of anticholinergic medications, which may influence systemic AChE and BChE levels. However, the use of point-of-care assessments allowed us to measure the current state of enzyme activity in the setting of critical illness, comorbid disease, and medication exposure to evaluate the associations of current functional levels with outcomes. This observational study cannot prove causation or direct mechanistic links between the enzymes and acute brain dysfunction, though we adjusted for a number of a priori identified confounders.

## Conclusions

We found that plasma cholinesterase activity is predictive of acute brain dysfunction during critical illness but not long-term impairments after discharge. Future studies should evaluate if cholinergic modulation in selected patients identified by plasma cholinesterase activity can reduce acute brain dysfunction.

## Supplementary Information

**Additional file 1.** Supplemental Appendix.

## Data Availability

All data generated or analyzed during this study are included in this published article and its Additional file. The datasets used and/or analyzed during the current study are available from the corresponding author on reasonable request.
