# Supplementary material for: Association between cholinesterase activity and critical illness brain dysfunction
Source: Crit Care. 2022 Dec 6;26:377. doi: 10.1186/s13054-022-04260-1 (PMC9724294; doi:10.1186/s13054-022-04260-1)
Supplement: Supplementary file 1 — Additional file 1. Supplemental Appendix. [file 13054_2022_4260_MOESM1_ESM.docx]

**Association between Cholinesterase Activity and Critical Illness Brain Dysfunction**

**SUPPLEMENTAL APPENDIX**

| **Additional Methods Information** | **2** |
| --- | --- |
| **References** | **3** |
| **Table S1. Characteristics of Patients with Long-term Follow-up** | **4** |
| **Table S2.** **Neuropsychological and Functional Assessments** | **5** |
| **Figure S1. Acetylcholinesterase Activity Levels During Hospital Stay** | **6** |
| **Figure S2. Acetylcholinesterase per Hemoglobin Activity Levels During Hospital Stay** | **7** |
| **Figure S3. Butyrylcholinesterase Activity Levels During Hospital Stay** | **8** |
| **Figure S4. Acetylcholinesterase Activity Levels and Mental Status** | **9** |
| **Figure S5. Acetylcholinesterase per Hemoglobin Activity Levels and Mental Status** | **10** |
| **Figure S6. Butyrylcholinesterase Activity Levels and Mental Status** | **11** |
| **Figure S7. Enzyme Activity Levels and Odds of Coma the Same Day** | **12** |
| **Figure S8. Enzyme Activity Levels and Odds of Delirium the Same Day** | **13** |
| **Figure S9. Enzyme Activity Levels and Odds of Increased Days Alive Without Delirium or Coma** | **14** |
| **Figure S10. Enzyme Activity Levels and Probability of Cognitive Impairment at Follow-up** | **15** |
| **Figure S11. Enzyme Activity Levels and Probability of Disability at Follow-up** | **16** |
| **Figure S12. Enzyme Activity Levels and Quality of Life at Follow-up** | **17** |
|  |  |
|  |  |
|  |  |
|  |  |
|  |  |
|  |  |
|  |  |

**Additional Methods Information**

Standard methods for detecting a minimum clinically important difference in neurocognitive scores typically suggest that changes exceeding 0.5 standard deviation constitute a meaningful difference.[1] We assessed cognitive function with the Repeatable Battery for the Assessment of Neuropsychological Status (RBANS)[2] and Trail Making Test Part B (Trails B)[3] or with the Telephone Interview for Cognitive Status (TICS)[4] and a validated telephone cognitive battery[5] depending on the co-enrolled study. The RBANS is a validated neuropsychometric battery that measures domains of attention, language, immediate and delayed memory, and visuospatial construction. The RBANS has a mean (standard deviation) population age-adjusted score of 100±15 with lower scores indicating worse global cognitive function. RBANS scores of 78 are representative of patients with traumatic brain injury and score of 70 representative of patients with mild Alzheimer’s disease.[2, 6] The Trails B[3] is a validated tool examining set shifting and cognitive flexibility. The Trails B has an age-, sex-, and education-adjusted mean score of 50±10 with lower scores indicating worse executive function. The Telephone Interview for Cognitive Status total score (TICS-T)[4] corrects for age since that may affect the performance of a population of individuals. Age-adjusted TICS-T scores have a potential range of 0 to 100, with a mean of 50±10, and normal scores typically fall between 40 and 60. Individuals earning TICS-T scores of ≤35 (1.5 standard deviations [SD] below the mean, ≤ 7th percentile) are considered to be cognitively impaired. The validated telephone cognitive battery[5] consisted of the Digit Span, Logical Memory I, Logical Memory II, Similarities, Controlled Oral Word Association, and Hayling Sentence Completion. We defined cognitive impairment present if the scores were ≥2 standard deviations below the population adjusted mean in one test (i.e., RBANS score ≤ 77.5, Trails B score ≤ 30, TICS-T score ≤35) or ≥1.5 standard deviations below the population adjusted mean in any two tests. We assessed basic ADLs and instrumental ADLs with the Katz ADL[7] and the Functional Activities Questionnaire (FAQ),[8] respectively. A score ≥ 1 on the ADL or ≥ 2 on the FAQ indicated disability in at least one domain of daily living. We assessed health-related quality of life with the EQ-5D questionnaire.[9] Values for the EQ-5D are anchored at 1 (full health) and 0 (a state as bad as being dead), with lower scores indicative of worse health-related quality of life.

**References**

1. Harvey PD: Clinical applications of neuropsychological assessment. *Dialogues Clin Neurosci* 2012, 14(1):91-99.

2. Randolph C, Tierney MC, Mohr E, Chase TN: The Repeatable Battery for the Assessment of Neuropsychological Status (RBANS): preliminary clinical validity. *J Clin Exp Neuropsychol* 1998, 20(3):310-319.

3. Reitan R, Wolfson D: The Halstead Reitan Neuropsychological Test Battery. Tuscon, AZ: Neuropsychology Press; 1985.

4. Brandt J, Spencer M, Folstein MF: The telephone interview for cognitive status. *Neuropsychiatry, Neuropsychology and Behavioral Neurology* 1988, 1(2):111-117.

5. Christie JD, Biester RC, Taichman DB, Shull WH, Jr., Hansen-Flaschen J, Shea JA, Hopkins RO: Formation and validation of a telephone battery to assess cognitive function in acute respiratory distress syndrome survivors. *Journal of critical care* 2006, 21(2):125-132.

6. McKay C, Casey JE, Wertheimer J, Fichtenberg NL: Reliability and validity of the RBANS in a traumatic brain injured sample. *Arch Clin Neuropsychol* 2007, 22(1):91-98.

7. Katz S, Ford AB, Moskowitz RW, Jackson BA, Jaffe MW: Studies of Illness in the Aged. The Index of Adl: A Standardized Measure of Biological and Psychosocial Function. *JAMA* 1963, 185:914-919.

8. Pfeffer RI, Kurosaki TT, Harrah CH, Jr., Chance JM, Filos S: Measurement of functional activities in older adults in the community. *J Gerontol* 1982, 37(3):323-329.

9. Rabin R, de Charro F: EQ-5D: a measure of health status from the EuroQol Group. *Ann Med* 2001, 33(5):337-343.

**Table S1. Characteristics of Patients with Long-term Follow-up**

| **Characteristic^*^** | **N=176** |
| --- | --- |
| Age at enrollment, years | 55 (38-65) |
| Education, N (%) |  |
| - Below bachelor’s degree | 144 (82%) |
| - Bachelor’s degree and above | 32 (18%) |
| Charlson Comorbidity Index at enrollment | 1 (0-2) |
| Disability present at enrollment, N (%) | 22 (13%) |
| Sepsis at enrollment, N (%) | 84 (48%) |
| ICU type, N (%) |  |
| - Medical | 45 (26%) |
| - Surgical | 45 (26%) |
| - Trauma | 86 (49%) |
| Days with mechanical ventilation† | 2.0 (0.0-5.0) |
| Days with delirium† | 2.0 (1.0-4.2) |
| Days with coma† | 0.0 (0.0-1.0) |
| Mean modified SOFA score‡ | 1.4 (0.7-2.5) |

^*^Median (interquartile range) or N (percentage).

† In the 14 days from enrollment

‡ Excluding the central nervous system component Glasgow Coma Scale

Participant characteristics and outcomes of patients in the cohort with long-term follow-up cohort are displayed.

Abbreviations: ICU, intensive care unit; SOFA, Sequential Organ Failure Assessment

**Table S2. Follow-up Assessments**

| **Assessment^*^** | **N** |  |
| --- | --- | --- |
| RBANS global score | 126 | 78 (67-85) |
| CLOX 1 score | 71 | 11 (9-12) |
| CLOX 2 score | 71 | 14 (13-14) |
| TRAILS A T-score | 126 | 42 (36-51) |
| TRAILS B T-score | 125 | 45 (36-52) |
| Hayling Test overall score | 23 | 5.0 (4.0-6.0) |
| Similarities scaled score | 26 | 8.0 (7.0-9.8) |
| Controlled Oral Word Association T-score | 25 | 39 (35-47) |
| Age-adjusted Digit Span score | 25 | 10 (7-12) |
| Logical Memory 1 score | 26 | 8.0 (5.2-9.8) |
| Logical Memory 2 score | 26 | 7.0 (5.2-10.8) |
| Telephone Interview Cognitive Status T-score | 27 | 42 (30-46) |
| Katz ADL score | 176 | 0 (0-1) |
| Functional Activities Questionnaire score | 29 | 6 (2-15) |
| Cognitive impairment present, N (%) | 154 | 72 (47%) |
| Disability present, N (%) | 176 | 71 (40%) |
| EQ-5D index score | 170 | 0.7 (0.4-0.8) |

^*^Median (interquartile range)

Results of the individual cognitive, disability, and health-related quality of life assessments are displayed. We defined cognitive impairment present if scores were ≥2 standard deviations below the population adjusted mean in one test (i.e., RBANS score ≤ 77.5, Trails B score ≤ 30, TICS-T score ≤35) or ≥1.5 standard deviations below the population adjusted mean in any two tests. We assessed basic ADLs and instrumental ADLs with the Katz ADL and the Functional Activities Questionnaire (FAQ), respectively. A score ≥ 1 on the ADL or ≥ 2 on the FAQ indicated disability in at least one domain of daily living.

Abbreviations: ADL, activities of daily living; RBANS, Repeatable Battery for the Assessment of Neuropsychological Status

**Figure S1. Acetylcholinesterase Activity Levels During Hospital Stay**

**
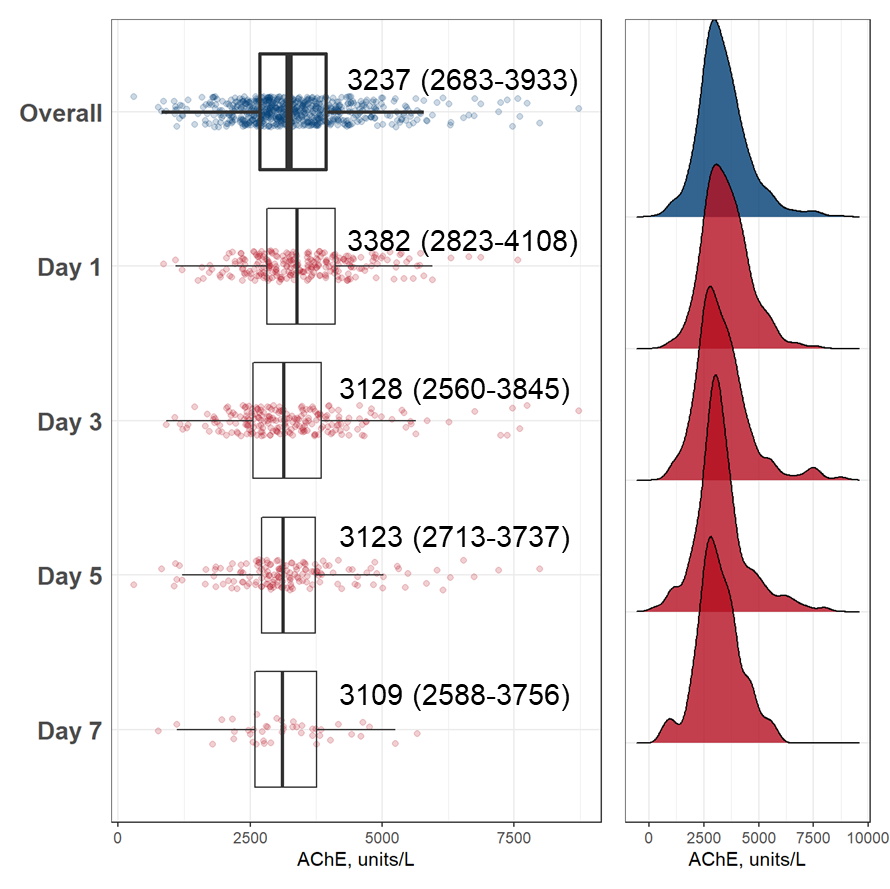
**

Acetylcholinesterase activity levels during the hospital stay are displayed. On the left, we present raw data (points) overlaid with summary measures (boxplots: middle line = median; leftmost line = 25^th^ percentile; rightmost line = 75^th^ percentile; extended lines cover all points within 1.5 x interquartile range of the median). The median (interquartile range) values are also displayed. On the right, we present density plots.

**Figure S2. Acetylcholinesterase per Hemoglobin Activity Levels During Hospital Stay**

**
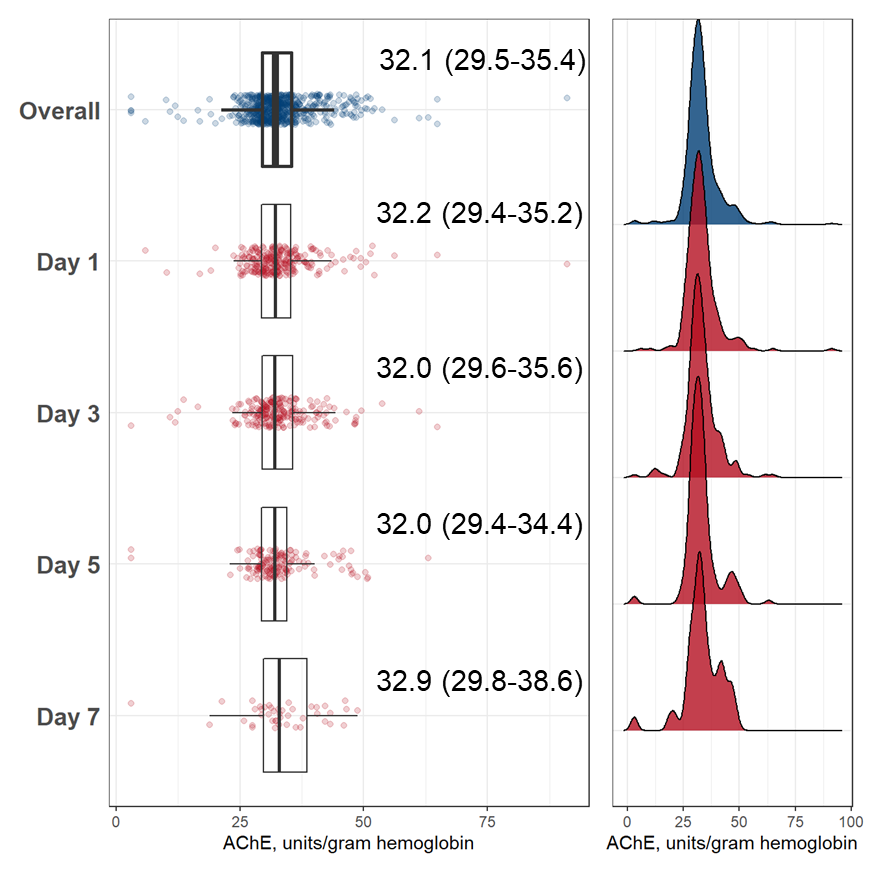
**

Acetylcholinesterase per hemoglobin activity levels during the hospital stay are displayed. On the left, we present raw data (points) overlaid with summary measures (boxplots: middle line = median; leftmost line = 25^th^ percentile; rightmost line = 75^th^ percentile; extended lines cover all points within 1.5 x interquartile range of the median). The median (interquartile range) values are also displayed. On the right, we present density plots.

**Figure S3. Butyrylcholinesterase Activity Levels During Hospital Stay**

**
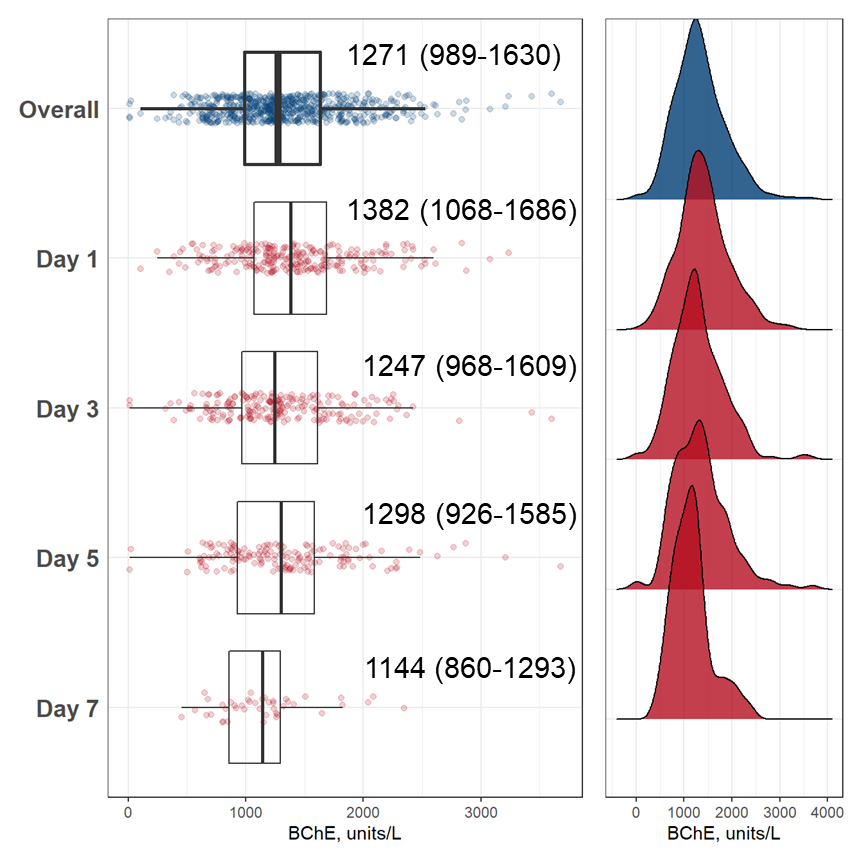
**

Butyrylcholinesterase activity levels during the hospital stay are displayed. On the left, we present raw data (points) overlaid with summary measures (boxplots: middle line = median; leftmost line = 25^th^ percentile; rightmost line = 75^th^ percentile; extended lines cover all points within 1.5 x interquartile range of the median). The median (interquartile range) values are also displayed. On the right, we present density plots.

**Figure S4. Acetylcholinesterase Activity Levels and Mental Status**

**
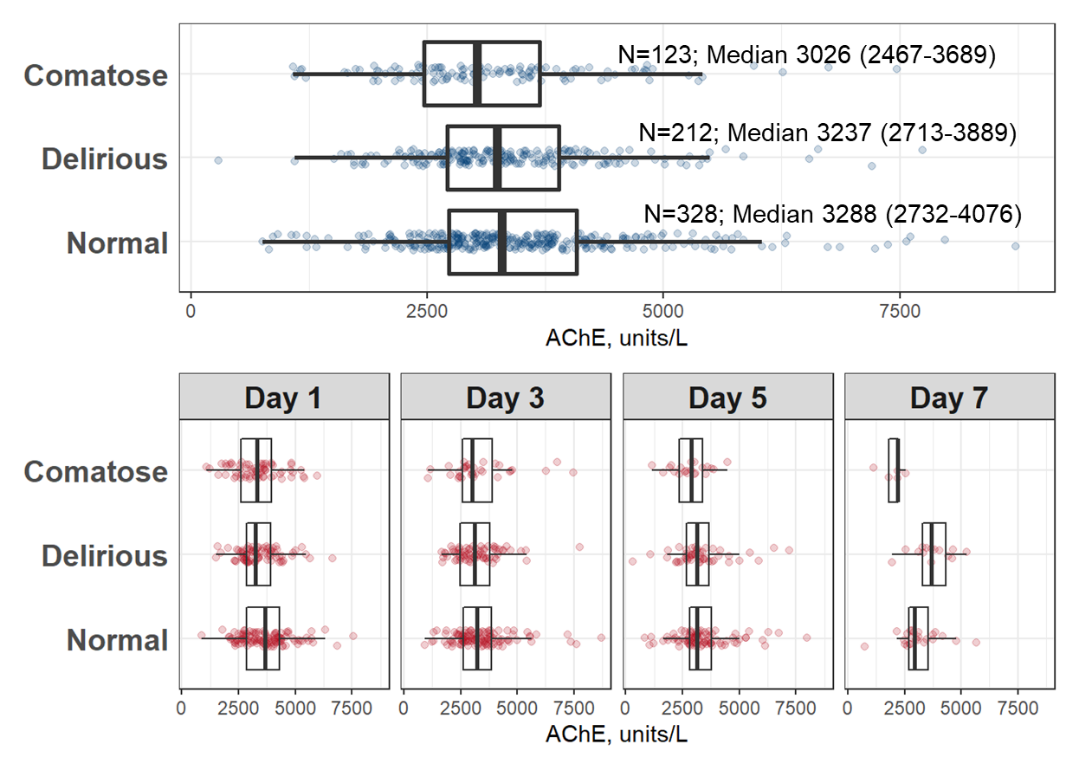
**

**
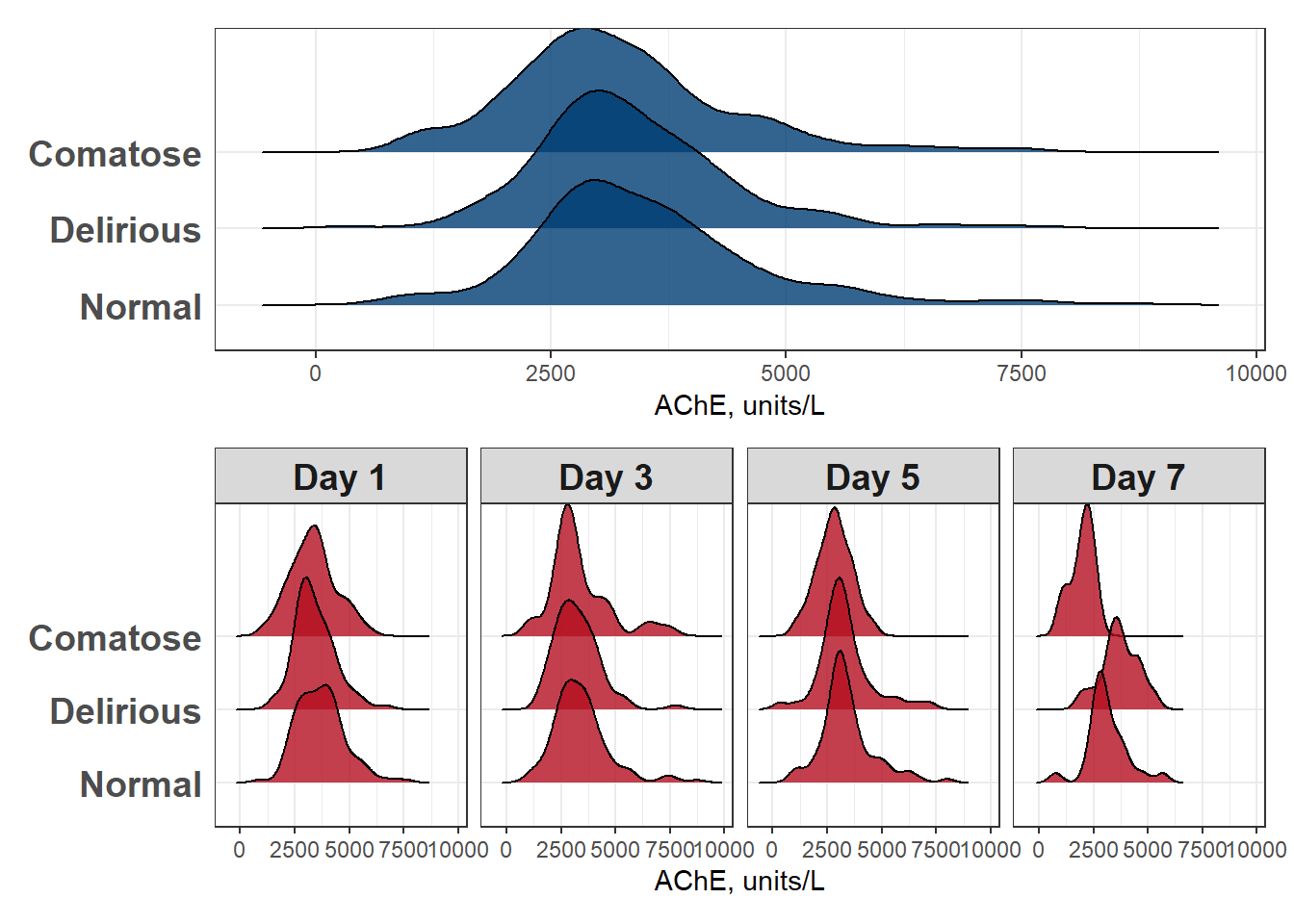
**

Acetylcholinesterase (AChE) activity levels on the days with normal, delirious, and comatose mental status are displayed. On the top panels, we present raw data (points) overlaid with summary measures (boxplots: middle line = median; leftmost line = 25^th^ percentile; rightmost line = 75^th^ percentile; extended lines cover all points within 1.5 x interquartile range of the median). On the bottom panels, we present density plots.

**Figure S5. Acetylcholinesterase per Hemoglobin Activity Levels and Mental Status**

**
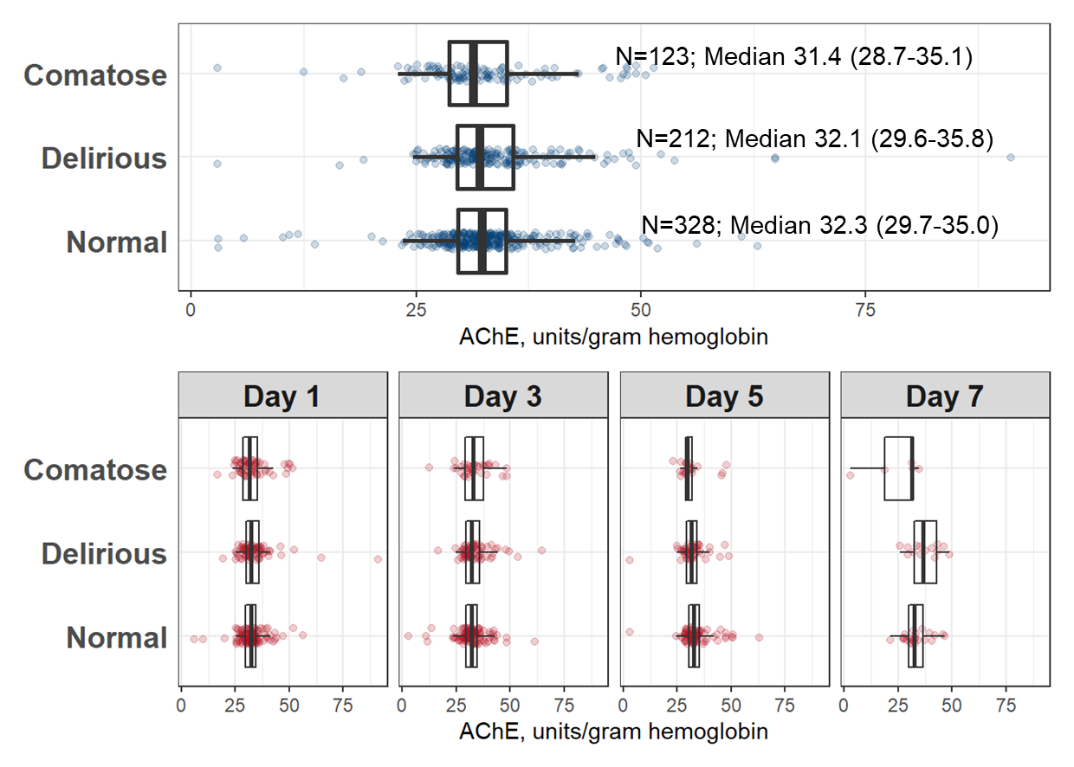
**

**
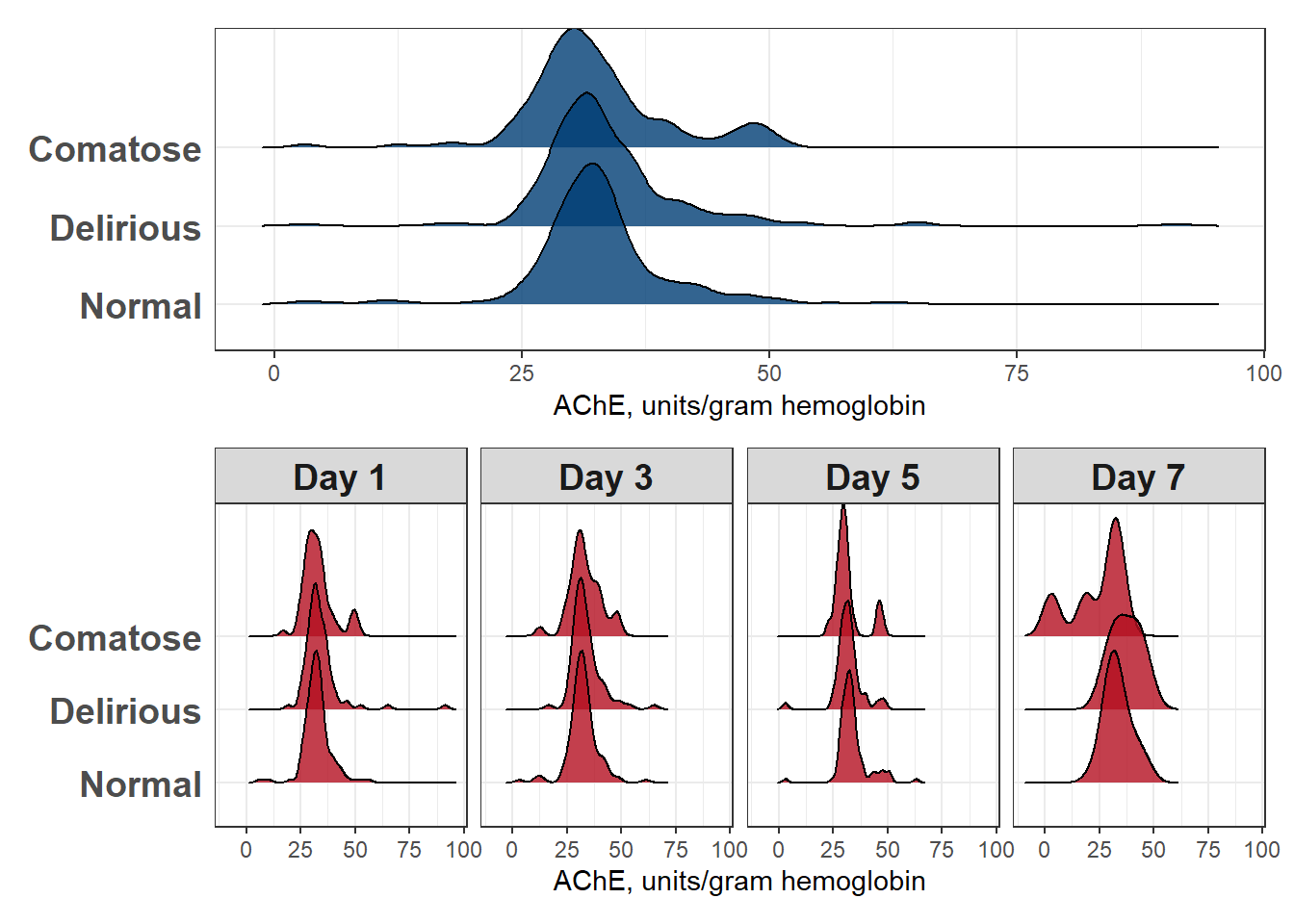
**

Acetylcholinesterase per hemoglobin (AChE/Hgb) activity levels on the days with normal, delirious, and comatose mental status are displayed. On the top panels, we present raw data (points) overlaid with summary measures (boxplots: middle line = median; leftmost line = 25^th^ percentile; rightmost line = 75^th^ percentile; extended lines cover all points within 1.5 x interquartile range of the median). On the bottom panels, we present density plots.

**Figure S6. Butyrylcholinesterase Activity Levels and Mental Status**

**
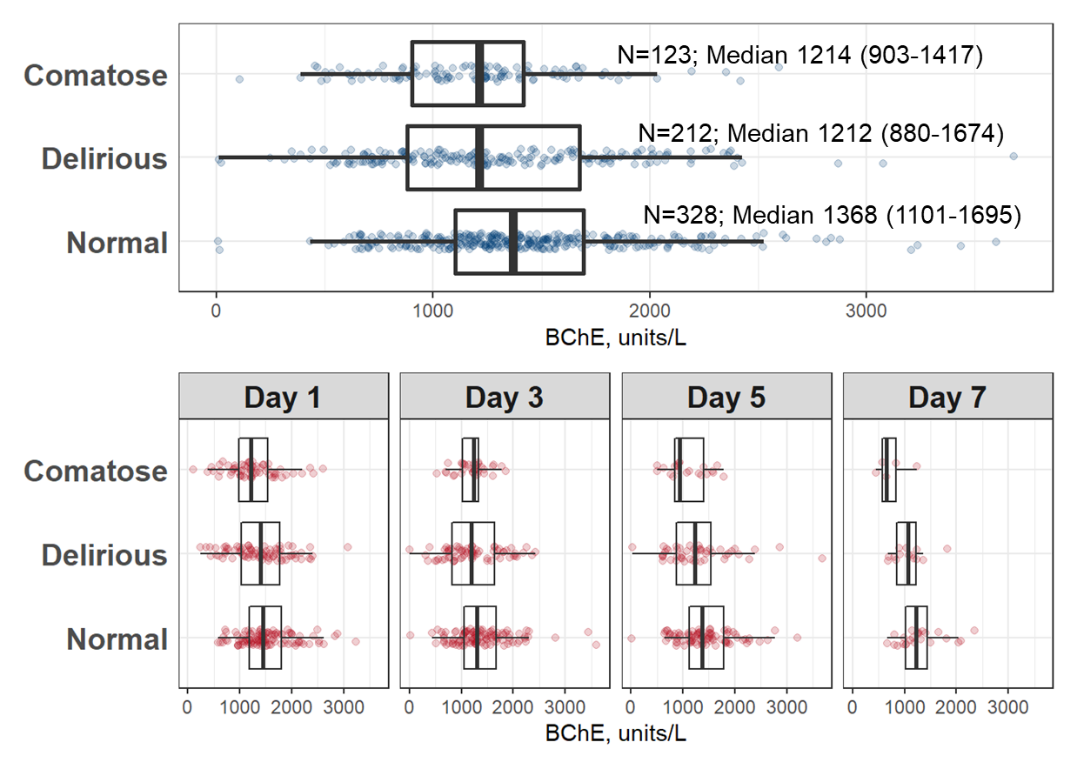
**

**
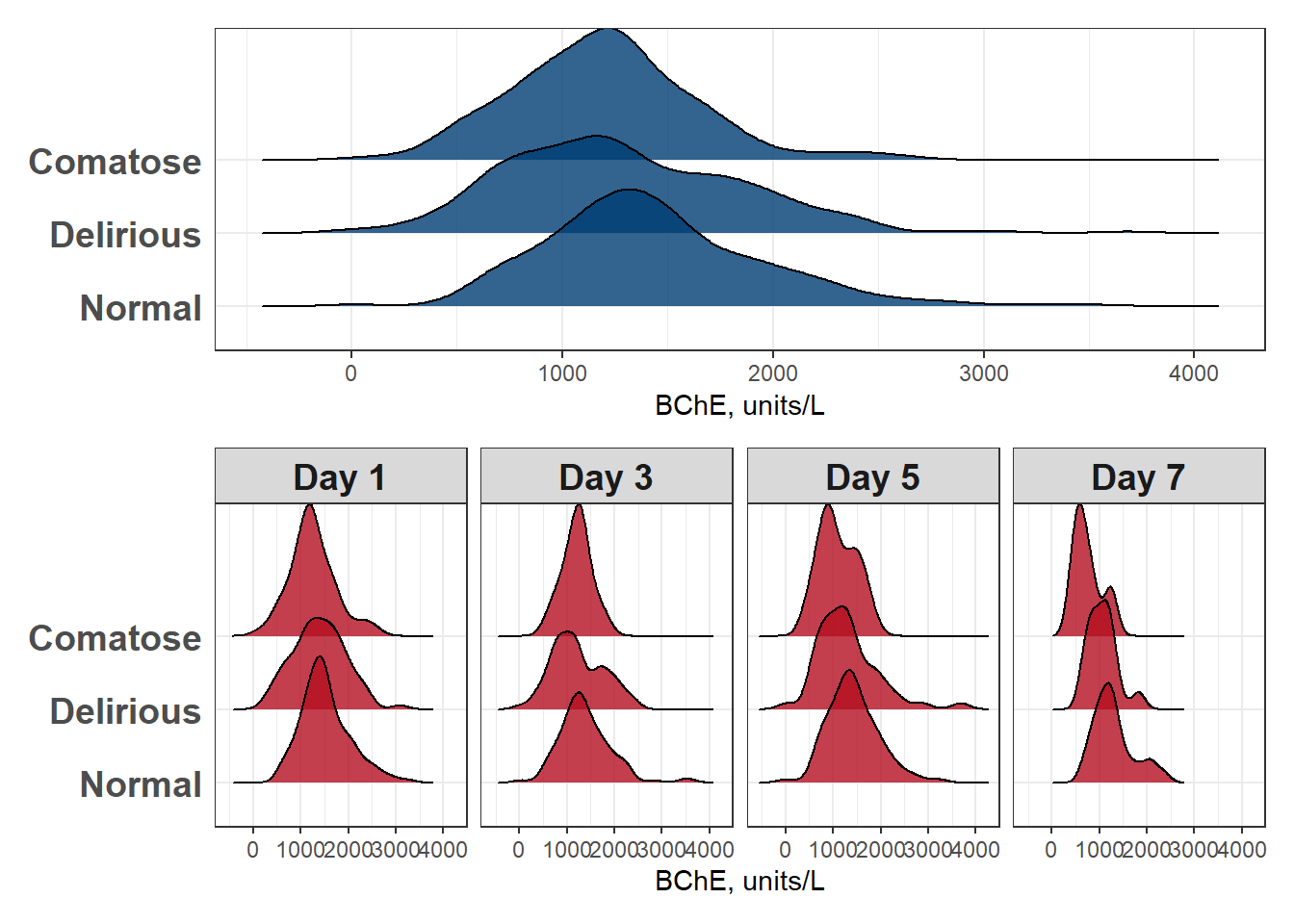
**

Butyrylcholinesterase (BChE) activity levels on the days with normal, delirious, and comatose mental status are displayed. On the top panels, we present raw data (points) overlaid with summary measures (boxplots: middle line = median; leftmost line = 25^th^ percentile; rightmost line = 75^th^ percentile; extended lines cover all points within 1.5 x interquartile range of the median). On the bottom panels, we present density plots.

**Figure S7. Enzyme Activity Levels and Odds of Coma the Same Day**

**
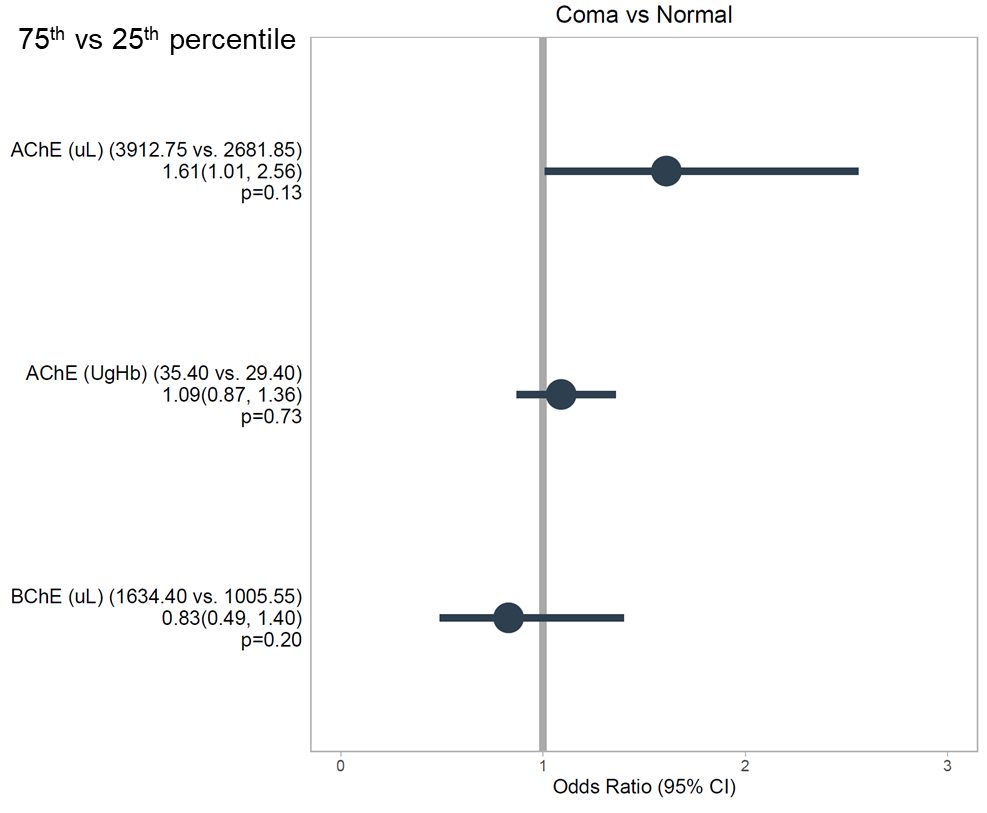
**

The odds of coma comparing the 75^th^ vs. 25^th^ percentile values of the enzyme activity within the cohort are displayed. Acetylcholinesterase (AChE), acetylcholinesterase per hemoglobin (AChE/Hgb), and butyrylcholinesterase (BChE) activity levels were not significantly associated with probability of comatose mental status.

**Figure S8. Enzyme Activity Levels and Odds of Delirium the Same Day**

**
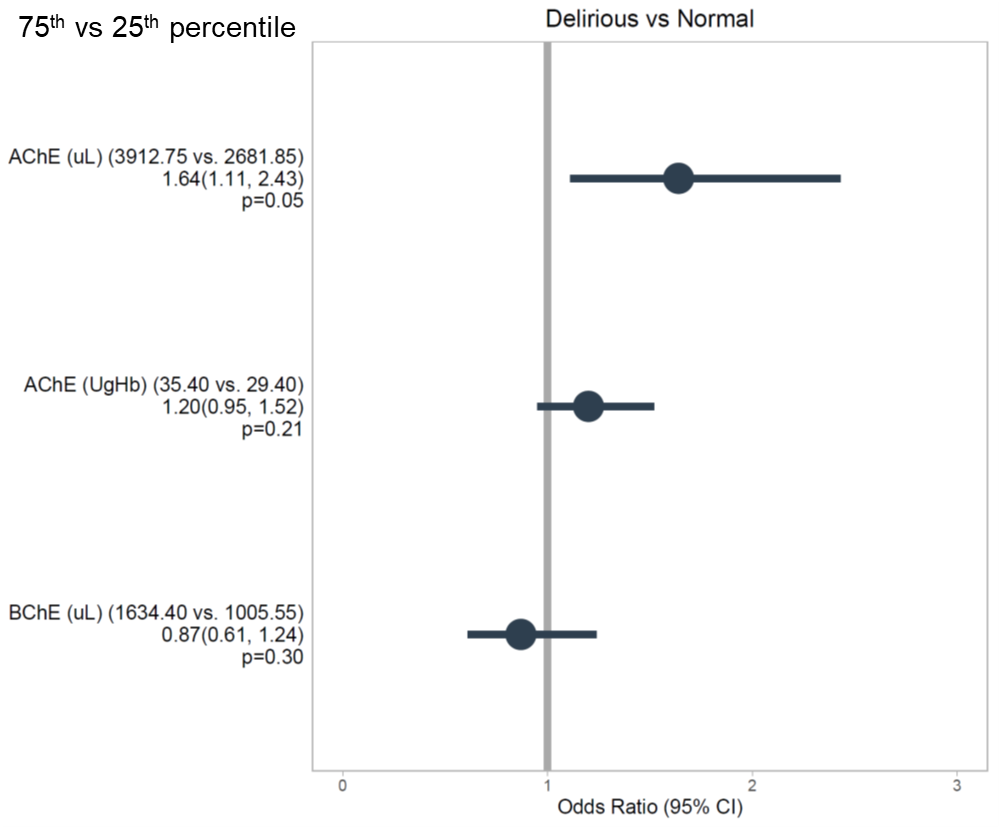
**

The odds of delirium comparing the 75^th^ vs. 25^th^ percentile values of the enzyme activity within the cohort are displayed. Acetylcholinesterase (AChE) levels were significantly associated delirious mental status (P=0.045). Patients in the 75^th^ percentile AChE level, for example, would have on average a 64% increased odds of having delirium compared to those in the 25^th^ percentile. Acetylcholinesterase per hemoglobin (AChE/Hgb) and butyrylcholinesterase (BChE) activity levels were not significantly associated with probability of delirious mental status.

**Figure S9. Enzyme Activity Levels and Odds of Increased Days Alive Without Delirium or Coma**

**
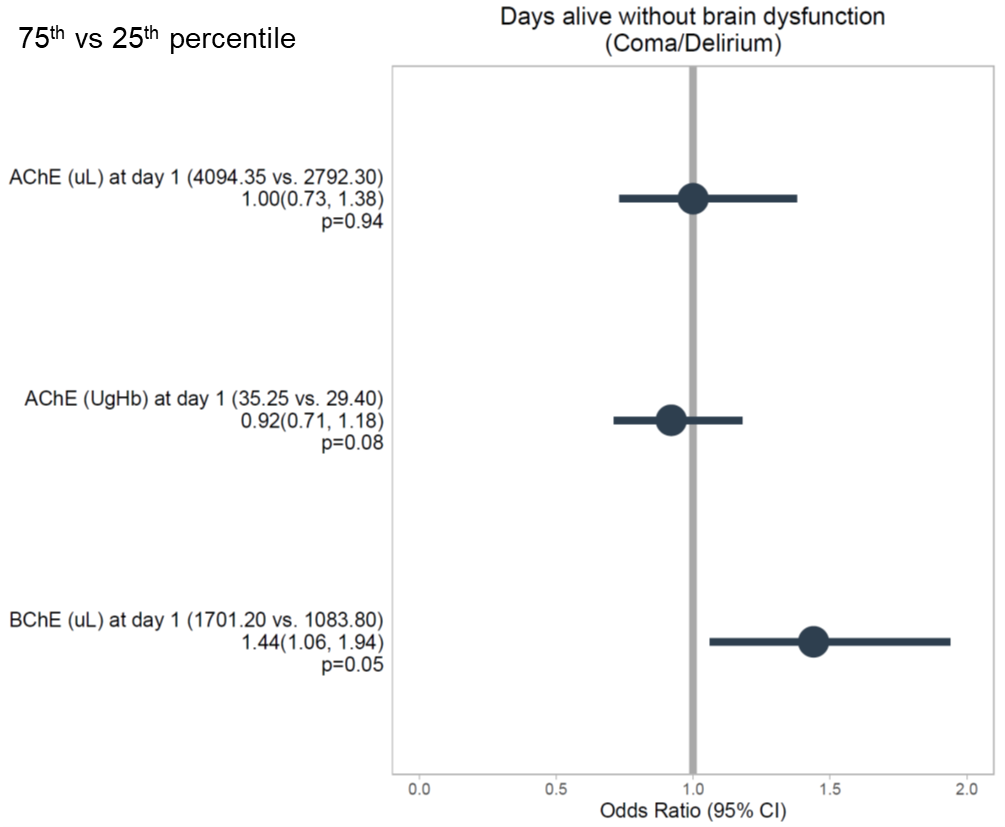
**

The odds of greater days alive without delirium or coma by the 75^th^ vs. 25^th^ percentile of the enrollment (day 1) enzyme values for the cohort are displayed. Lower enrollment BChE activity levels were associated with fewer days alive without delirium or coma over the following 14 days (P=0.048), indicating worse acute brain dysfunction. Patients in the 75^th^ percentile of BChE activity, for example, would have on average a 44% increased odds of having more days alive without delirium or coma (favorable outcome, indicating less brain dysfunction) compared to those in the 25^th^ percentile, supporting that lower BChE values are associated with worse acute brain dysfunction. Enrollment AChE and AChE/Hgb activity were not significantly associated with days alive without delirium or coma.

**Figure S10. Enzyme Activity Levels and Probability of Cognitive Impairment at Follow-up**

**
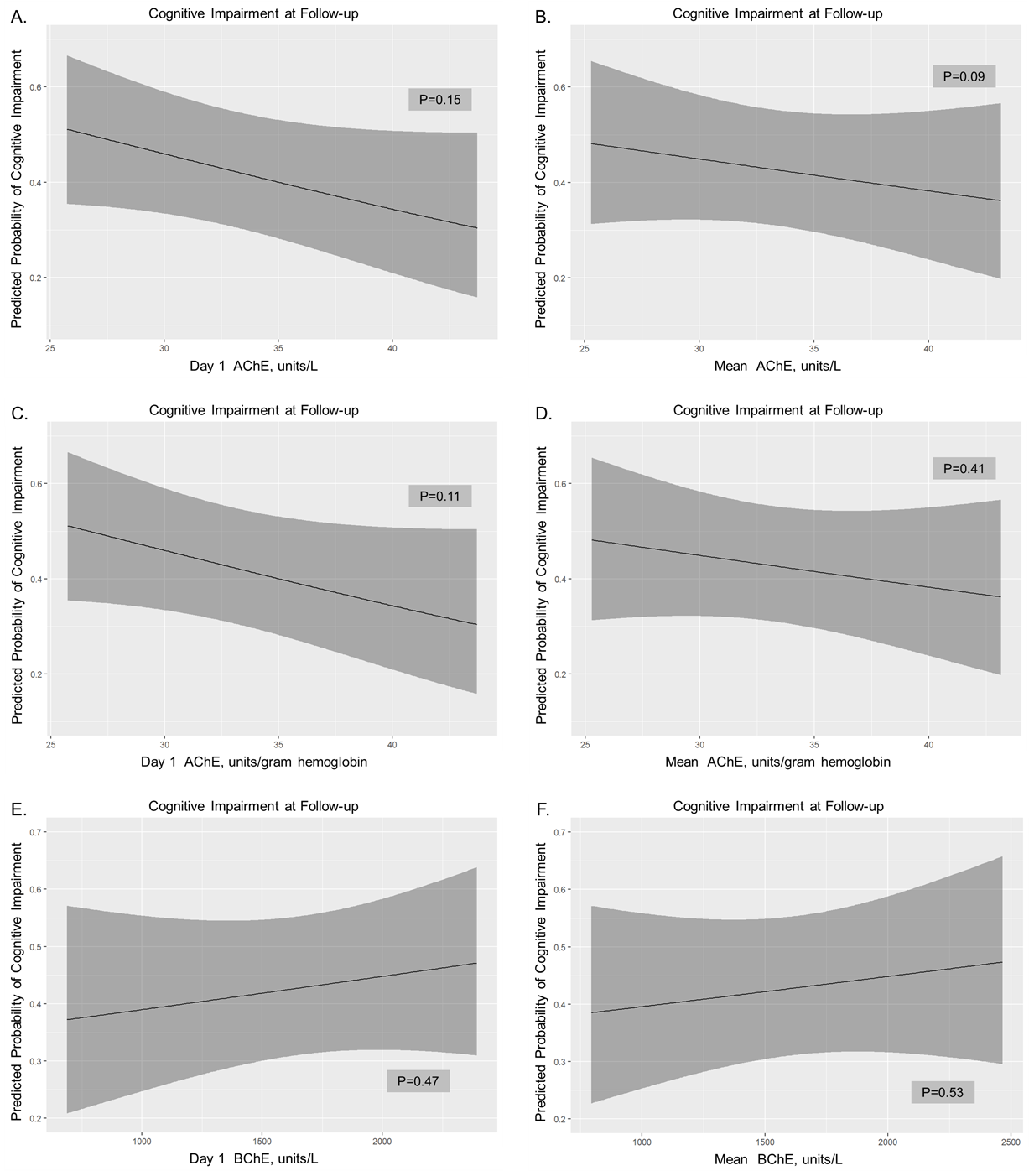
**

The enzyme activity levels at enrollment (day 1, panels A, C, E) and mean activity levels (panels B, D, F) and their associations with cognitive impairment are displayed. We did not find any statistically significant associations between either enrollment or mean acetylcholinesterase (AChE), acetylcholinesterase per hemoglobin (AChE/Hgb), or butyrylcholinesterase (BChE) activity levels and cognitive impairment up to 6 months after discharge.

**Figure S11. Enzyme Activity Levels and Probability of Disability at Follow-up**

**
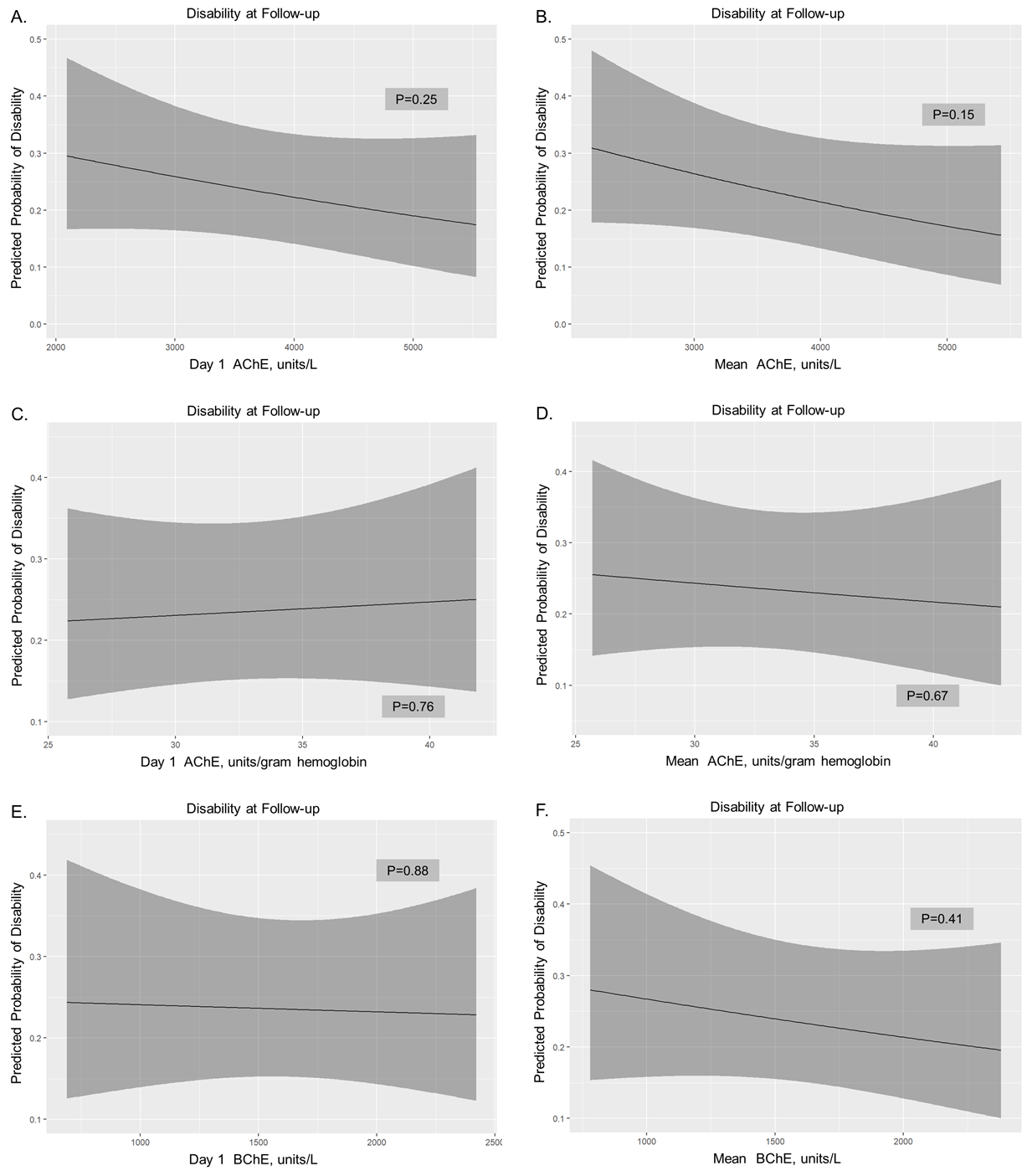
**

The enzyme activity levels at enrollment (day 1, panels A, C, E) and mean activity levels (panels B, D, F) and their associations with disability are displayed. We did not find any statistically significant associations between either enrollment or mean acetylcholinesterase (AChE), acetylcholinesterase per hemoglobin (AChE/Hgb), or butyrylcholinesterase (BChE) activity levels and disability up to 6 months after discharge.

**Figure S12. Enzyme Activity Levels and Health-Related Quality of Life at Follow-up**

**
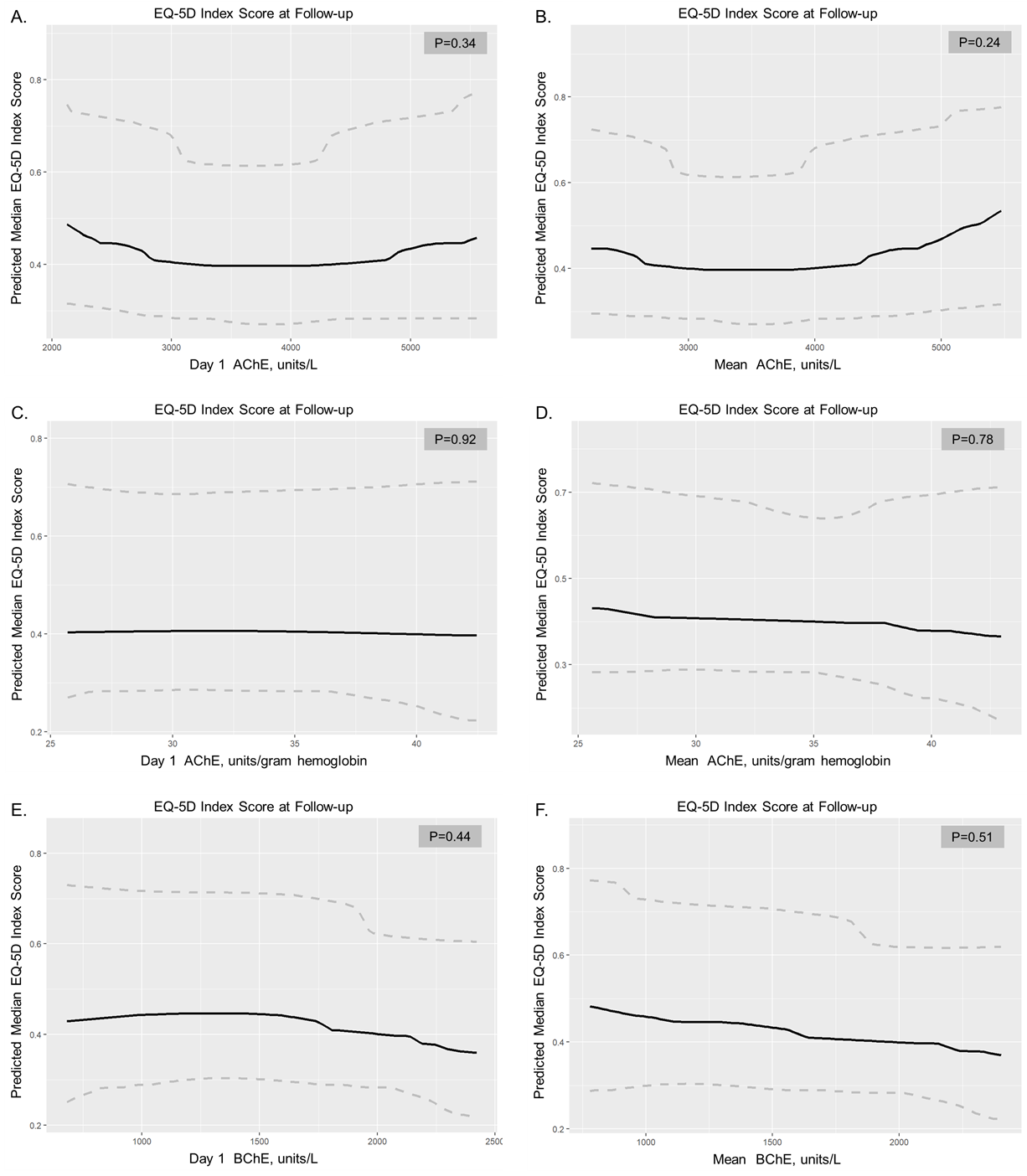
**

The enzyme activity levels at enrollment (day 1, panels A, C, E) and mean activity levels (panels B, D, F) and their associations with EQ-5D index scores are displayed. We did not find any statistically significant associations between either enrollment or mean acetylcholinesterase (AChE), acetylcholinesterase per hemoglobin (AChE/Hgb), or butyrylcholinesterase (BChE) activity levels and health-related quality of life up to 6 months after discharge.
